# Iron deficiency and depression: evidence of critical risk periods

**DOI:** 10.3389/fpsyt.2026.1770460

**Published:** 2026-02-04

**Authors:** Ravi Philip Rajkumar

**Affiliations:** Department of Psychiatry, Jawaharlal Institute of Postgraduate Medical Education and Research, Pondicherry, India

**Keywords:** depression, ferritin, ferroportin, hepcidin, iron deficiency, iron deficiency anemia, postpartum depression

## Introduction

Iron deficiency anemia (IDA) and depressive disorder – also known as major depressive disorder (MDD) or simply “depression” – are among the leading causes of disability worldwide (1). The possibility of an association between these two seemingly unrelated conditions has been considered for over half a century. In 1973, Fischbach and colleagues found evidence of reduced serum iron in women with MDD, which was associated with reduced gastric acid secretion. They suggested that reduced gastric acidity could lead to impaired iron absorption, which could alter catecholamine metabolism and trigger episodes of depression (2, 3). Though this hypothesis has not been confirmed, several researchers have documented significant links between IDA and MDD (4, 5). In some cases, iron deficiency appears to be associated depression even in the absence of anemia, suggesting a pathophysiological link between iron metabolism and depression (6, 7). However, other researchers have failed to replicate these results (8, 9), and it is unclear whether iron deficiency plays a causal role in MDD, or *vice versa* (10).

A portion of the variability seen across studies can be explained in terms of methodological differences, including differences in study population, in the use of depressive symptoms or MDD as the outcome of interest, and in the cut-off values used to define iron deficiency or IDA (11, 12). However, an alternative way of interpreting the available data is that there are certain periods of the human life cycle when iron deficiency is likely to play a role in the pathogenesis of depression. This paper provides a brief outline and analysis of the evidence for this contention. Before appraising this evidence, it is important to briefly review the neurobiological links between iron deficiency and MDD.

## Iron metabolism and depression

Iron is the most abundant of the “trace elements” found in the brain, with particularly high concentrations observed in the basal ganglia and the substantia nigra of the midbrain. Besides its role as an essential constituent of hemoglobin, enabling oxygen transport to the brain, iron also plays an important role in the synthesis of myelin and of monoamine neurotransmitters such as serotonin and dopamine (13). Free iron also interacts with monoamine oxidase isoforms, which catabolize these neurotransmitters (14). However, an excess of cerebral iron can lead to oxidative stress, neuronal damage, and even cell death via the process of ferroptosis (15). The protein ferritin plays a central role in maintaining iron homoeostasis in the brain, and the hormone hepcidin regulates iron levels by facilitating its transport to the extracellular space via the protein ferroportin (13, 16). An optimal level of cerebral iron is essential for brain development as well as for normal cognitive and emotional functioning (17). A recent study using mouse models found that both chronic stress and iron deprivation could induce cerebral iron deficiency, causing reduced neurogenesis and glucocorticoid receptor dysfunction in the hippocampus, which led to depressive-like behaviors (18).

These results support the notion that cerebral iron levels are implicated in the pathophysiology of depression. Additional support comes from studies of patients with MDD or IDA. Over 50% of patients with patients with IDA may have depressive symptoms severe enough to qualify for a diagnosis of MDD (19). In patients with MDD and comorbid IDA, the severity of iron deficiency is positively correlated with the severity of depressive symptoms in both community and hospital settings (20, 21). The association between these two conditions does not appear to be simply caused by the poor appetite or self-neglect sometimes seen in depression: only about 6-9% of patients with depression have a dietary iron intake below the average requirement (22).

How can this evidence be reconciled with the mixed results of human studies on the prevalence of depression in persons with iron deficiency? A possible answer can be arrived at by examining the results of pre-clinical and clinical research on the multiple factors influencing both cerebral iron levels and their relationship with depression (10, 14, 18). This answer is at least partially corroborated by the existing literature, which suggests that the association between iron deficiency and depression is particularly strong at three periods of life: (i) adolescence, (ii) pregnancy and the puerperium, and (ii) old age. These periods share three important common features. First, they are associated with an increased vulnerability to depression *per se*, independent of iron or other nutrient status. Second, they are associated with an increased risk of iron deficiency, symptomatic or otherwise. Third, they are characterized by increased levels of physiological and psychological stress, which interact with – or even trigger – abnormalities in brain iron metabolism, thereby increasing the risk of depression. It is, of course, possible that other such periods exist, but the available evidence for these three is the strongest, and will be outlined below. This evidence, presented in a narrative format due to its heterogeneity, was obtained from searches of the PubMed, Scopus, and ScienceDirect databases, using the terms “iron deficiency”, “iron deficiency anemia”, and their variants along with “depression”, “major depression”, “depressive disorder” and their variants, for a period up to and including November 2025. All relevant original research retrieved using these searchers, either in humans or in animal models, was consulted in the preparation of this paper.

Before examining the literature connecting iron deficiency to depression in these populations, it is helpful to briefly review the laboratory assessment of iron deficiency and IDA, as well as the associations between peripheral and central iron levels.

## Measuring iron deficiency

IDA is diagnosed based on the presence of (a) anemia, based on sex-specific hemoglobin (Hb%) cut-offs, and (b) biochemical evidence of iron deficiency. Within cells, iron is stored bound to ferritin, while in peripheral blood, it is transported by the binding protein transferrin. **Table 1** provides a summary of the peripheral measures of iron deficiency and their strengths and weaknesses (23). Most significantly, these measures may be unreliable in the presence of infection, inflammation, cancer, or certain chronic medical diseases. This should be taken into account when evaluating studies using these markers, particularly in the elderly.

**Table 1:** Laboratory measures of iron deficiency.

Measure	Physiological significance	Change observed in IDA	Limitations
Serum ferritin	Binds iron intracellularly Acute-phase protein	↓	Increases during infection, inflammatory disorders, and certain malignancies, even in the presence of iron deficiency
Serum iron	Direct measure of circulating iron	↓	Also reduced in anemia secondary to chronic medical illness.
Serum transferrin	Binds and transports iron in peripheral blood	↑	Can be reduced or normal in inflammatory disorders, even if there is iron deficiency.
Transferrin saturation	Measures the amount of iron bound to transferrin	↓	Also reduced in anemia secondary to chronic medical illness
TIBC	Measures the maximum amount of iron that can be bound to transferrin	↑	Can be reduced or normal in inflammatory disorders, even if there is iron deficiency.
Ferroportin	Transports iron out of cells	Not routinely measured	Reduced during infection or inflammatory disorders.
Hepcidin	Binds to ferroportin and inhibits release of intracellular iron	Not routinely measured	Expression increased during infection or inflammatory disorders.

IDA, iron-deficiency anemia; ↑, increased; ↓, decreased; TIBC, total iron-binding capacity.

It should also be noted that these measures of iron deficiency are not directly correlated with cerebral iron levels. In a study using a cell culture model, it was found that serum ferritin was not significantly associated with serum-to-cerebrospinal fluid (CSF) iron transport (24). Currently, iron is considered to enter the brain through detachment from circulating transferrin and transport across the blood-brain barrier (BBB) via endothelial cells; however, the exact relationships between peripheral transferrin or TIBC and cerebral iron are not fully understood (25). Moreover, iron transport at the BBB may be influenced by factors such as sex, hepcidin levels, and variants in other genes linked to brain iron transport (26, 27). Thus, even if a study demonstrates biochemical evidence of peripheral iron deficiency in association with depression, it is not clear to what extent this truly reflects cerebral iron deficiency or dysregulation.

## Iron deficiency and depression in adolescence

Iron is essential in brain development and synaptic plasticity (28), and maternal iron deficiency has been associated with adverse neurodevelopmental outcomes, such as delays in the acquisition of language and motor skills (29, 30). In healthy infants without IDA, iron supplementation at ages 6–12 months was associated with significant increases in positive affect, cooperativeness, task persistence, and stress tolerance at 10 years of age (31). Inadequate body iron stores – and, by extension, reduced cerebral iron availability – could be associated with behavioral and emotional deficits that increase the risk of depression in later childhood or adolescence.

Studies from both developed and developing countries are consistent with this conclusion. In adolescents from Sudan, serum ferritin – a peripheral marker of iron stores – was significantly reduced in those with MDD. There was a negative correlation between serum ferritin and the severity of depressive symptoms (32), similar to observations in adults with MDD (20). A case-control study from Switzerland found no difference in serum ferritin, but reduced soluble transferrin receptor levels, in adolescents (age 13-17) with MDD. Adolescents with MDD were also more likely to report being diagnosed with iron deficiency in this sample (33). Evidence of an association between peripheral markers of iron deficiency and brain structure was obtained from a study of adolescent girls (aged 12-17) with depressive (73%) or anxiety disorders and no prior exposure to psychotropic medication. In these patients, serum ferritin was negatively correlated with the severity of both depression and anxiety. Additionally, serum ferritin was negatively correlated with the volume of the left caudate nucleus and bilateral putamen, suggesting a link between iron deficiency and alterations in brain structure and connectivity in this age group (34). From a therapeutic perspective, a pilot study of school-going adolescents (age 16–19 years) suggests that iron supplementation in those with IDA was associated with a significant reduction in self-reported depression. This was temporally associated with normalization of serum ferritin (35). Anecdotal evidence suggests that adolescents with IDA and depressive symptoms do not respond adequately to antidepressants, and may benefit from iron replacement (36).

Similar results were obtained in two large datasets based on general population data. In an analysis of national health insurance data from Taiwan, involving 2957 children and adolescents with IDA and matched control, this diagnosis was associated with a two-fold increase in the risk of depression. IDA was also associated with bipolar disorder in this sample, but only in girls (37). In a similar study using data from the United States’ National Health and Nutrition Examination Survey (NHANES), both IDA and iron deficiency without anemia were associated with depressive symptoms in adolescents who identified as multiracial (38). This association remained significant even after adjustment for possible confounders.

The available evidence suggests that iron deficiency – clinical or subclinical – may be associated with an increased risk of depression in adolescence. Preliminary evidence of altered basal ganglia structure has been demonstrated in this context. The available data also suggests that this association could be influenced by factors such as gender and ethnicity (37, 38). It is highly probable that other moderating factors exist, including genetic vulnerability to depression and adverse experiences in childhood or adolescence (39). Iron deficiency dating back to early childhood can impair the acquisition of skills protective against depression (31), which may lead to an increased sensitivity to adverse life events in adolescence.

## Iron deficiency and peripartum depression

Iron deficiency is common during pregnancy, and may be due to prior nutritional deficits, increased physiological requirements during fetal development, or both (40). These requirements increase over the course of pregnancy, and are compounded by the blood loss that occurs during childbirth. As a result, studies examining the association between IDA and depression in early pregnancy have been inconclusive (41, 42), while those focused on the third trimester or postpartum period have generally yielded positive results (40).

Studies from developed countries have found biochemical evidence of iron deficiency in 30-40% of pregnant women. Iron deficiency has been associated with a 2.5-fold increase in depression risk in the third trimester of pregnancy, and with an increase in depressive symptom severity from the second trimester to the first postpartum month (5, 43, 44). Conversely, higher peripheral levels of the iron-related biomarkers ferritin and hepcidin have been associated with fewer depressive symptoms in the third trimester (45). Lower serum ferritin 48 hours after birth is also significantly associated with postpartum depressive symptoms 2 and 8 months later; overall, low serum ferritin was associated with a nearly four-fold increase in subsequent postpartum depression (46). In low-income or low-resource settings, iron deficiency anemia is three times more common in women with PPD, and iron deficiency has been linked to the co-occurrence of PPD and cognitive impairment (47, 48). Lower serum iron levels may also interact with reduced levels of neuroactive steroids, such as allopregnanolone, to influence depression risk during pregnancy (49).

A recent study of a rodent model of postpartum depression suggests that iron supplementation during pregnancy may be as effective as the antidepressant fluoxetine in reducing depressive-like behavior, preventing neuronal loss, and increasing neural plasticity (50). As yet, there is insufficient evidence that routine iron supplementation prevents PPD in women without iron deficiency (51, 52). A recent study has found that intravenous iron supplementation was associated with a significant improvement in depressive symptom scores over oral iron supplements, regardless of hemoglobin level (53). another study, involving women with anemia of any cause (Hb < 11 g/dL), found a trend towards improved depressive symptoms as anemia improved with iron replacement. However, the study was underpowered to address this question (54).

The above results notwithstanding, it should be acknowledged that some researchers have failed to find an association between iron deficiency and peripartum depression (55). Moreover, perinatal depression is not simply the result of iron or other nutrient deficiency: it arises from an interaction between genetic vulnerability, the physiological stressors associated with pregnancy and childbirth, and a wide range of psychosocial factors, including marital disharmony, socioeconomic disadvantage, and a lack of social support (10). These factors are associated not just with depression but with iron deficiency, leading to significant confounding effects (56). Intravenous iron preparations may be more effective than oral supplements at correcting both anemia and depression, though this has not yet been confirmed (57). Despite the provisional nature of the available evidence, it remains plausible that iron deficiency is associated with an elevated risk of peripartum depression, and that iron supplementation may relieve depression in these women through putative direct effects on brain functioning (50, 58). Such treatment should be combined with appropriate psychosocial interventions, which may improve both maternal nutrition and mood (56).

## Iron deficiency and late-life depression

The elderly population of the world is growing steadily in most countries, and this age group is at an elevated risk of depression compared to the general population. Recent evidence suggests that untreated depression in late life is a modifiable risk factor for dementia (59). In this context, it is worth noting that iron deficiency and IDA are both very common in the elderly: about 20-30% of adults aged over 65 may have iron deficiency (60, 61). Iron deficiency in the elderly is multifactorial: it may be due to nutritional deficits, blood loss, adverse effects of medications (such as aspirin or proton pump inhibitors) or even the aging process itself (62–64). IDA in the elderly is associated with significant reductions in physical activity, which is itself a risk factor for depression (65).

Research on associations between iron deficiency and geriatric depression, particularly from a mechanistic perspective, is still in its early stages. A large population-based study found that both anemia and low serum ferritin were associated with depressive symptoms in adults aged 65 and above (66). A smaller study of adults aged 55 and above found that nearly 30% had iron deficiency. In these adults, indices of iron storage and red cell production were positively correlated with performance on standardized cognitive tests; on the other hand, depression was negatively correlated with measures of iron transport and saturation. Fatigue was also associated with measures of iron deficiency (67). These results suggest that iron deficiency may affect both mood and cognitive function in older adults; this is of particular interest in view of the known link between depression and dementia.

To date, no study has directly investigated alterations in brain functioning in relation to iron deficiency in living elderly adults. Recent results suggest that hepcidin and ferroportin play key roles in regulating cerebral iron levels in this age group (62, 68). In a study of samples obtained from a brain bank, observed patterns of mRNA expression revealed reduced expressions of heavy ferritin and ceruloplasmin in white matter lesions. This pattern was suggestive of increased cellular efflux and reduced influx of iron (69). As white matter lesions are associated with both depression and cognitive impairment in the elderly (70), this finding supports the hypothesis that cerebral iron metabolism is altered in geriatric depression. However, it is not clear how this result can be linked to iron deficiency.

## Interactions between iron deficiency and other risk factors for depression

Depression is a disorder of multifactorial origin, arising from interactions between genetic vulnerability – itself polygenic in nature – and multiple risk or protective factors (71). This must be appreciated when examining the links between iron deficiency and depression: instead of a simple linear model, low peripheral or central iron is one of many interacting variables contributing to depression risk. While a comprehensive review of the environmental risk factors for depression is beyond the scope of this paper, key factors in the three populations being considered – children and adolescents, women in the peripartum period, and the elderly – are summarized below in **Table 2** (72–74**).**

**Table 2:** Environmental risk factors for depression in specific populations.

Population	Risk factors
Children and adolescents (72)	• Female gender • Hormonal changes related to puberty • Early parental separation or loss • Childhood abuse or neglect • Socioeconomic disadvantage • Lifestyle factors (diet, sleep, exercise) • Substance use (tobacco, alcohol, cannabis, others) • Age-specific stressors (e.g., peer-related, academic, bullying) • Comorbid chronic medical illness
Peripartum women (73)	• Prior medical illness • Past history of depression • Lack of social support • Unplanned or unwanted pregnancy • Marital discord and intimate partner violence • Socioeconomic disadvantage • Hormonal changes related to pregnancy and childbirth • Pregnancy and birth-related complications (e.g., pregnancy-induced hypertension, gestational diabetes mellitus, prolonged labor, postpartum hemorrhage)
Elderly adults (74)	• Female gender • Past history of depression • Physical frailty • Disability • Cognitive impairment • Bereavement • Chronic or multiple medical comorbidities • Use of multiple medications

Some of the factors enumerated are above are significant because they represent risk factors not only for depression but for IDA. In some cases, such as postpartum hemorrhage, this link is obvious; in others, such as polypharmacy in the elderly, the association is more subtle. This becomes important when attempting to model the associations between iron deficiency and depression in these populations: demonstrating a simple linear association or correlation may not be a valid approach, and more advanced models, such as multivariate regression or structural equation modeling, may be required to identify mediation or moderation effects. For example, socioeconomic disadvantage may influence depression risk both by increasing exposure to chronic stressors and through an increased risk of nutritional iron deficiency.

An additional consideration in this regard is the existence of gender differences in the risk of both depression and IDA. This may be of particular concern in adolescents, where hormonal and social vulnerabilities towards depression (75) interact with an increased risk of iron deficiency through menstrual bleeding, even in apparently healthy girls (76).

## Critical evaluation of the available data

**Figure 1:**
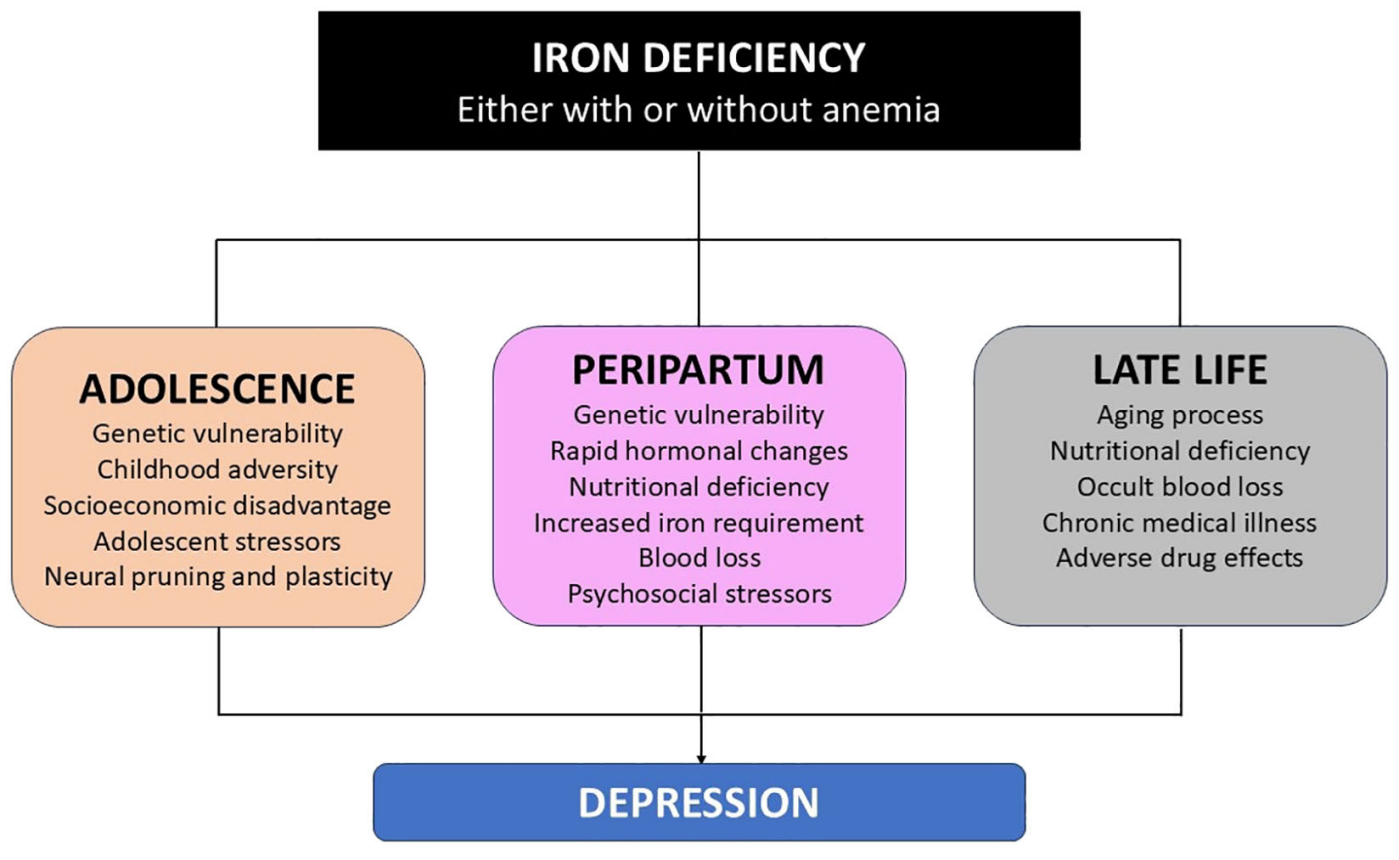
Association between iron deficiency and depression in vulnerable populations.

A careful examination of the available data suggests that iron deficiency is a potential risk factor for depressive symptoms and MDD in adolescence and in women in the third trimester of pregnancy and postpartum. The evidence for such an association in the elderly is weaker, but still suggestive. These findings are in contrast to the inconsistent results in general population samples, and suggest that iron deficiency may be more likely to trigger or maintain depression when it occurs in association with other biological or psychosocial risk factors. Such factors are particularly operative at these three phases of the human life cycle. An illustration of this hypothesis is presented in **Figure 1**.

The findings discussed above should be contrasted with recent evidence that an excess – rather than a deficit – of cerebral iron may be associated with depression. In a mouse model, inactivation of the ferroportin 1 (*FPN1*) gene in oligodendrocytes led to depressive-like behaviors. These behaviors were associated with disruptions in myelin structure, increased intracellular iron, oxidative stress, and neuroinflammation through the interleukin-6 (IL-6) pathway (77). Likewise, in a rat model of depression induced by chronic stress, hepcidin expression and iron levels in the hippocampus were increased; this was associated with elevated serum IL-6, and could be blocked by the administration of the hepcidin antagonist dalteparin (78). Subsequently, studies using magnetic resonance imaging (MRI) to estimate tissue iron levels *in vivo* have found evidence of elevated iron in specific brain regions, particularly the putamen and thalamus, in patients with depression. These findings are not associated with systemic iron deficiency or excess. Instead, they appear to be caused by increased central hepcidin expression, brain inflammation, and iron deposition in affected regions (79–81). It is unknown if such changes play a primary role in the pathogenesis of depression, or if they are secondary to other mechanisms. The most parsimonious interpretation of these findings is that cerebral iron levels are tightly regulated in relation to optimal mental functioning, and that both a deficit and an excess can lead to depression, albeit through different pathways (68).

Certain limitations of the available literature must be acknowledged. First, most available research is cross-sectional or retrospective in nature. Therefore, the prospective nature of the link between iron deficiency and depression requires confirmation, particularly in adolescents and in the elderly, through the use of longitudinal cohort studies or biobank data. Second, most research did not account for confounding variables, particularly for the psychosocial factors that often trigger depression in these three populations; this has been discussed in more depth in the preceding section. Third, despite some clues from animal and human research, the exact mechanisms through which iron deficiency can cause depression remain to be elucidated. Fourth, it is unknown if iron deficiency is specifically associated with depression, to other mood disorders such as bipolar disorder, or with a general increase in susceptibility to several types of mental illness (37). Fifth, the possibility of reverse causality cannot be ruled out: depression may increase the risk of iron deficiency, particularly in older adults, due to reduced dietary intake or systemic inflammation (82). Finally, due to the diverse and heterogeneous nature of the existing data, a more formal synthesis could not be undertaken: this should prove possible with future growth of research in this area.

## Conclusions

Iron deficiency may be a risk factor for depression in adolescents, women in the peripartum period, and the elderly. In the elderly, iron deficiency may also be associated with concurrent cognitive impairment. Iron deficiency is not the sole cause of depression in these groups, but acts in concert with other age-specific biological and psychosocial factors. Possible mechanisms linking iron deficiency and depression include reduced neural plasticity, neuroendocrine dysregulation, alterations in regional brain structure, and altered levels of cerebral iron regulators such as hepcidin and ferroportin. The evidence summarized in this paper provides a starting point for future research on the mechanisms linking iron deficiency with depression, and the interactions between iron deficiency and other established risk factors for depression. From a research perspective, the existing literature highlights the need for more precise and cutting-edge assessments of peripheral and cerebral iron metabolism in patients from these vulnerable groups suffering from depression. The former would entail measurements of markers such as ferritin, hepcidin and ferroportin in peripheral blood; the latter could involve more extensive use of MRI protocols that assess iron deposition across brain regions. Additionally, future research in this field would benefit from methodological and statistical approaches that can identify possible mediation or causality more clearly. From a clinical perspective, once these findings are confirmed, there is a need for more consistent assessment of biochemical markers of iron deficiency in patients with depression belonging to these groups, keeping in mind the other factors that can affect these markers. In adolescents and postpartum women, iron supplementation may prove to be a useful adjunctive strategy to ameliorate depression when there is an incomplete response to antidepressants. In elderly persons with depression and biochemical evidence of iron deficiency, it may be helpful to address secondary causes of iron deficiency, including blood loss and medications.
